# No association between telomere length and osteonecrosis of the femoral head

**DOI:** 10.1186/s12891-021-04047-5

**Published:** 2021-02-12

**Authors:** Si-Wook Lee, Kyung-Hwan Lim, Kyung-Jae Lee, Yu-Ran Heo, Jae-Ho Lee

**Affiliations:** 1 Department of Orthopaedic Surgery, Keimyung University School of Medicine, Daegu, Republic of Korea; 2 Department of Anatomy, Keimyung University School of Medicine, Daegu, Republic of Korea

**Keywords:** Telomere length, ONFH, Osteonecrosis, Erythrocyte sedimentation rate, Femoral head

## Abstract

**Background:**

Telemore length (TL) shortening has been found in many diseases. However, clinical characteristics of TL shortening in osteonecrosis of the femoral head (ONFH) has not been investigated. Therefore, we studied whether TL changes have clinicopathological values in ONFH.

**Methods:**

The TL in the synovial tissues of 36 ONFH and 127 control patients (femoral neck fracture) was examined by quantitative real-time PCR as relative length, Δ Ct value. In addition, the correlation between TL and clinical features of ONFH and controls was analyzed.

**Results:**

The average TL in the femoral tissues was 1.46 ± 3.12 (standard deviation). The average TL in the ONFH and control tissues was 1.92 ± 4.11 and 1.34 ± 2.78, respectively, however, the difference was absent (*p* = 0.324). Furthermore, a shorter TL was tended to be associated with erythrocyte sedimentation rate (100% vs. 61.5%, *p* = 0.073); however, the association was not statistically significant.

**Conclusions:**

In this study, we demonstrated that there is no association between the TL and clinicopathologic characteristics of ONFH patients. However, further studies considering the genetic factors are needed to be performed.

## Background

Telomeres are specific structures positioned at the end of chromosomes that together with specific protein complexes bound to them, provide DNA protection, and ensure genomic stability [1]. Telomeric repeats are lost during every cell division, because of lacking replication of the 3′-end of the chromosome. It induces critically short telomeres, occurring cellular senescence or crisis [2]. Telomere length (TL) declines progressively with aging. However, this decline is apparently accelerated in the presence of oxidative stress and inflammation. The TL is determined at birth in individuals and reflects the lifelong cumulative burden effect of inflammation and oxidative stress [3, 4].

Osteonecrosis of the femoral head (ONFH) is a disease involving death of the cells of the femoral head, with subsequent structural changes, leading to progressive collapse of the femoral head by degenerative arthritis in hip joint [5]. In South Korea, its prevalence was over 20 per 100,000 people in 2002 and 14,103 per year on average [6]. Its prevalence was 1.4 to 3.0 per 100,000 in UK and 1.9 per 100,000 in Japanese [7]. Although the exact pathology of ONFH has not been fully clarified, several causes of ONFH have been suggested, such as microvessel destruction, fat embolism due to altered lipid metabolism, and increased bone marrow pressure with fat cell enlargement [8–10]. Furthermore, oxidative stress and altered metabolism caused by various etiological factors, such as corticosteroids, alcohol abuse, radiation, and Gaucher disease, are thought to be a risk factor for the development of ONFH [11]. Aging may be a key factor in ONFH pathogenesis; though, there was only few genetic studies on TL in ONFH. Many surgical studies has been performed for ONFH, molecular diagnosis and treatment was not focused [12, 13]. Moreover, the synovial tissues of ONFH patients have not been used, thus, limiting our efforts to clarify the pathogenesis of the disease. Therefore, TL analysis of ONFH may provide new information for its pathogenesis and clinical characteristics.

In the present study, TL in synovial tissues of ONFH and femoral head fracture patients (control) was studied, and then correlated it with the clinicopathological characteristics of the patients.

## Methods

### Patients and DNA extraction

A total of 163 patients, who underwent femoral head surgery for the treatment of ONFH or femoral head fracture at the Keimyung University Dongsan Hospital from September 2009 to October 2011, were included in the current study. The surgeons explained the purpose of the study to the patients and obtained informed consent from all participant. The Institutional Review Board of Keimyung University Dongsan Medical Center approved the study (IRB No. DSMC-2016-01-041-001). All the patients with ONFH or traumatic femoral neck fracture were diagnosed independently by two orthopedists and pathologist. The synovial tissues were provided by The Keimyung Human Bio-resource Bank in Korea, which were frozen in liquid nitrogen and stored at − 80 °C. Patients with severe chronic diseases, such as cancer, cardiovascular diseases, immunodeficiency virus infection, diabetes mellitus, and renal dysfunction were excluded.

Genomic DNA from the synovial tissues was extracted using the QIAamp DNA mini kit (Qiagen, Inc., Valencia, CA, USA). The quantity and quality of the extracted DNA was measured using a NanoDrop 1000 spectrophotometer (Thermo Scientific, Wilmington, DE, USA).

### Telomere length analysis

The TL of each chromosome was analyzed by quantitative real-time polymerase chain reaction (qPCR). To analyze quantitative TL relative to nuclear DNA (S), primers for assessing the TL were selected using specific amplification (T) and ß-globin primers were used for nuclear DNA (S), according to a previous study [14]. qPCR was performed using the LightCycler 480 II system (Roche Diagnostics, Mannheim, Germany). TL was presented as T/S values and calculated as follows:

$$ \mathrm{T}/\mathrm{S}=2-\Delta  \mathrm{Ct} $$

where ∆Ct = average Ct telomere - average Ct ß-globin. Each measurement was performed in triplicate and five serially diluted control samples were included in each experiment.

### Statistical analysis

The SPSS statistical package (version 25.0, Windows) was used for all the statistical analyses. TL was represented as the mean ± standard deviation. In addition, Chi-square test, Mann–Whitney U test, and simple correlation analysis were performed for analysis. A *p*-value of < 0.05 was considered to be statistically significant.

## Results

**Fig. 1:**
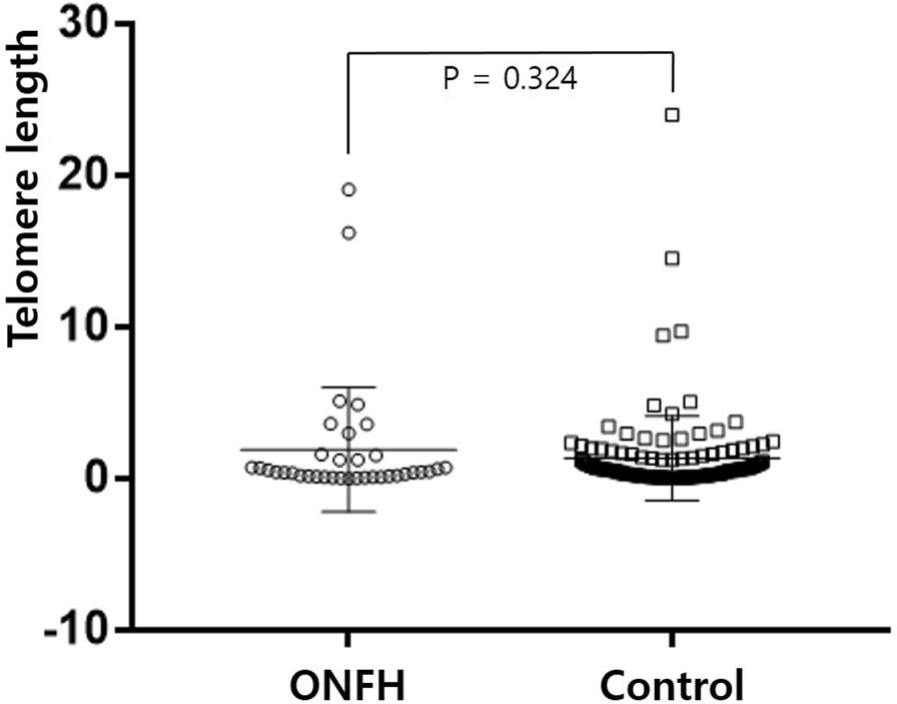
Telomere length in osteonecrosis of the femoral head (ONFH) and control (femoral head fracture) patients

The average age of the 163 patients was 62.8 years (26–93 years). There were 74 (43.5%) male and 89 (52.4%) female. According to the diagnoses by orthopedists, there were 36 (22.1%) ONFH and 127 (77.9%) femoral head fracture patients. The qPCR analysis indicated the average TL to be 1.46 ± 3.12 in the femoral tissues. Furthermore, the average TL was found to be 1.92 ± 4.11 and 1.34 ± 2.78 in ONFH and femoral head fracture (control) patients, respectively. As shown in Fig. 1, the difference in the TL was not statistically significant (*p* = 0.324) between the ONFH and control groups.

Furthermore, to clarify the clinical value of TL in ONFH, the patients were divided into two groups based on the average value of TL. Among all the patients, 52 (31.9%) exhibited longer TL, while 111 (68.1%) exhibited shorter TL. Table 1 summarizes the clinicopathological parameters of the femoral head injury patients, including the ONFH and fracture groups based on TL. Our results showed that none of the variables had any association with the TL. The clinicopathological characteristics of ONFH patients based on TL are represented in Table 2. A shorter TL was found to be associated with erythrocyte sedimentation rate (ESR, 100% vs. 61.5%, *p* = 0.073); however, it was statistically insignificant. The other clinicopathological characteristics were not associated with the TL.

**Table 1:** Telomere length in synovial tissue of femoral head injury

	Telomere length (%, n)		
	Short	Long	***p***
All patients	68.1 (111)	31.9 (52)	
Disease			0.844
ONFH	69.4 (25)	30.6 (11)	
Fracture	67.7 (86)	32.3 (41)	
Age			0.294
< 65	71.8 (61)	28.2 (24)	
≥ 65	64.1 (50)	35.9 (28)	
Gender			0.379
Male	71.6 (53)	28.4 (21)	
Female	65.2 (58)	34.8 (31)	
Side			0.223
Right	67.1 (49)	32.9 (24)	
Left	64.8 (46)	35.2 (25)	
Both	90.0 (9)	10.0 (1)	
ESR			0.232
(+)	80.0 (16)	20.0 (4)	
(−)	66.7 (88)	33.3 (44)	
CRP			0.359
(+)	60.7 (17)	39.3 (11)	
(−)	69.7 (85)	30.3 (37)	
Alcohol			0.620
(+)	70.8 (17)	29.2 (7)	
(−)	65.6 (84)	34.4 (44)	

*ONFH* Osteonecrosis of the femoral head, *ESR* Erythrocyte sedimentation rate, CRP:

**Table 2:** Telomere length in the synovial tissue of ONFH

	Telomere length (%, n)		
	Short	Long	***p***
All patients	69.4 (25)	30.6 (11)	
Age			0.418
< 65	75.0 (15)	25.0 (5)	
≥ 65	62.5 (10)	37.5 (6)	
Gender			0.669
Male	66.7 (14)	33.3 (7)	
Female	73.3 (11)	26.7 (4)	
Side			0.210
Right	61.5 (8)	38.5 (5)	
Left	66.7 (10)	33.3 (5)	
Both	100 (6)	0 (0)	
ESR			**0.073**
(+)	100 (7)	0 (0)	
(−)	61.5 (16)	38.5 (10)	
CRP			0.151
(+)	33.3 (1)	66.7 (2)	
(−)	73.3 (22)	26.7 (8)	
Cause			0.813
Steroid	60.0 (3)	40.0 (2)	
Alcohol	73.7 (14)	26.3 (5)	
Idiopathic	66.7 (8)	33.3 (4)	
Alcohol			0.467
(+)	73.7 (14)	26.3 (5)	
(−)	61.5 (8)	38.5 (5)	

**Fig. 2:**
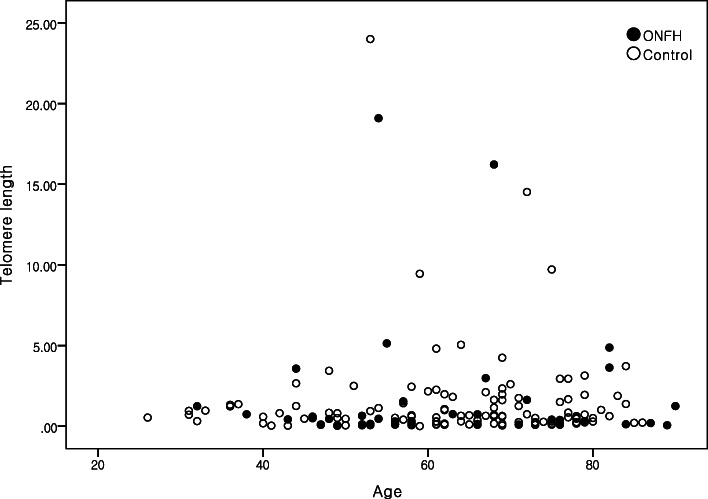
Telomere length is not correlated with age in the patients with osteonecrosis of the femoral head (ONFH) and fracture (control)

*ONFH* Osteonecrosis of the femoral head, *ESR* Erythrocyte sedimentation rate, CRP:

Thus, the quantitative analysis showed no correlation between the TL and clinical values in patients with ONFH and femoral head injury. Furthermore, as shown in Fig. 2, TL was not correlated with the age of the patients (*r* = 0.012, *p* = 0.876).

## Discussion

To the best of our knowledge, we analyzed and compared the differences in TL between ONFH and femoral neck fracture patients for the first time. Most studies on TL used the peripheral blood samples from patients with cancer, diabetes, and psychological diseases [15, 16]; however we used DNA extracted from the synovial tissues of ONFH and femoral neck fracture. TL is a putative marker of biological aging and has been shown to be reduced in patients with age-related diseases, such as Alzheimer’s disease, dementia, atherosclerosis, and hypertension [3, 17, 18]. Furthermore, several studies have suggested that TL is reduced in inflammatory diseases, such as rheumatoid arthritis [4]. This decline by oxidative damage may be due to the following reasons: (1) chronic inflammation, (2) increased ROS production, (3) increased base oxidation, (4) persistent damage to telomeres, and (4) an increased rate of telomere shortening [19]. Therefore, we hypothesized that change in TL may play an important role in ONFH pathogenesis.

Our results showed no difference in TL between the ONFH and control subjects. Furthermore, previous result has showed an inverse correlation between TL and age [20], though, our results were in disagreement with previous findings. In addition, since TL varies between the individuals at birth, it may be difficult to demonstrate a causal relationship between age and TL in human population studies, without taking the genetic factors into account. Because most patients in present study were old, especially ONFH, age distribution was not even. This limitation may affects correlation between TL and age. Hence, further studies should be performed in ONFH patients with large number of cases.

Interestingly, a shorter TL tended to be associated with ESR in ONFH. ESR increases in cases of inflammation, infections, anemia, autoimmune disorders, and other inflammatory conditions [21]. ESR is frequently high in ONFH because of the inflammatory processes, however, it is also nonspecific [20]. This suggests that ONFH may be associated with telomere shortening caused by inflammatory processes. However, its detailed molecular mechanisms should be studied further.

## Conclusions

We identified a low clinicopathological value of TL in the synovial tissues of ONFH. However, its association with inflammatory markers provides a new clue for ONFH pathogenesis. TL may be affected by lifestyle, such as smoking, nutrition, and air pollution, as well as socioeconomic factors and psychological stress. Therefore, further studies should identify the role of telomere regulation in larger cases of ONFH and its therapeutic possibility should be discussed.

## Data Availability

The datasets used during the present study are available from the corresponding author on reasonable request.

## Abbreviations

ONFH: Osteonecrosis of the femoral head
TL: Telemore length
qPCR: Quantitative real-time polymerase chain reaction
